# Medical Device-Associated Infections in Intensive Care Settings: Biofilms, Multidrug-Resistant Pathogens, and Prevention Strategies

**DOI:** 10.7759/cureus.111893

**Published:** 2026-07-01

**Authors:** Anam A Fakir, Satish R Patil, Priyanka M Mane

**Affiliations:** 1 Department of Microbiology, Krishna Vishwa Vidyapeeth (Deemed to Be University), Karad, IND

**Keywords:** antimicrobial resistance (amr), biofilm, catheter-associated urinary tract infections (cautis), central line-associated bloodstream infections (clabsi), device-associated infections, healthcare-associated infections (hais), infection prevention, intensive care unit (icu), multidrug-resistant pathogens, ventilator-associated pneumonia (vap)

## Abstract

A large percentage of healthcare-associated infections (HAIs) are medical device-associated infections (DAIs), which significantly raise patient morbidity, mortality, and medical costs. The use of invasive medical devices such as ventilators, central venous catheters, and urinary catheters is linked to a significant number of HAIs. Reports from the World Health Organization and extensive epidemiological studies highlight the pervasive effects of HAIs, particularly in resource-constrained environments. Ventilator-associated pneumonia, central line-associated bloodstream infections, and catheter-associated urinary tract infections are the most common forms of medical DAIs. These infections frequently result in longer hospital stays and more expensive medical care. Longer device use and inadequate aseptic procedures increase the risk of infection. Furthermore, because implant-related infections are recurrent and persistent, they present additional treatment challenges. One of the main causes of medical DAIs is biofilm formation. Biofilms are structured groups of microorganisms encased in an extracellular matrix that enhances their survival and resistance. Adhesion, maturation, and dispersion are the stages of biofilm formation that lead to persistent infections. These biofilms offer defense against the immune system and antibiotics. *Staphylococcus aureus*, *Pseudomonas aeruginosa*, *Acinetobacter baumannii*, and *Klebsiella pneumoniae* are common pathogens linked to DAIs; many of these pathogens exhibit multidrug resistance (MDR). Mechanisms such as carbapenem resistance and extended-spectrum β-lactamase production complicate treatment even more. On its priority list, the World Health Organization emphasizes the urgent need to address MDR pathogens.

## Introduction and background

Since the advent of invasive medical procedures and implantable medical devices in contemporary medicine, healthcare-associated infections (HAIs) have been acknowledged as a significant issue. The 20th century saw an increase in the use of prosthetic joints, arterial catheters, urinary catheters, and mechanical ventilation systems, which led to the first reports of device-related infections (DAIs). Subsequent research revealed that microorganisms could survive on device surfaces through biofilm development, resulting in chronic and recurrent infections [1,2]. Initial investigations mostly concentrated on contamination during insertion procedures. Biofilms are organized communities of microorganisms enclosed within a self-produced extracellular polymeric substance matrix that adheres to living or inert surfaces [3]. This discovery significantly advanced the understanding of medical DAIs by explaining why many infections persist despite appropriate antibiotic therapy.

The frequency of DAIs has dramatically grown over the past few decades due to the rapid expansion of intensive care units, prolonged hospital stays, organ transplantation, and the increasing use of implantable prosthetic devices [1,4]. Concurrently, the overuse and misuse of broad-spectrum antibiotics hastened the emergence of extensively drug-resistant (XDR) and multidrug-resistant (MDR) pathogens, making clinical management even more challenging [5-7]. Historically, bacteria such as coagulase-negative staphylococci and *Staphylococcus aureus* were considered the primary causes of device-associated infections. However, current epidemiological trends indicate an increasing involvement of Gram-negative bacteria, including *Pseudomonas aeruginosa*,* Acinetobacter baumannii*,* and Klebsiella pneumoniae*. Many of these pathogens possess the ability to form biofilms and exhibit carbapenem resistance, meaning they are resistant to carbapenems, a class of last-resort antibiotics used to treat severe bacterial infections [8-11]. Some of these organisms also produce extended-spectrum β-lactamases (ESBLs), enzymes that break down many commonly used β-lactam antibiotics, further limiting treatment options. Due to these developments, medical DAIs have become a major global healthcare concern. The understanding and management of medical DAIs have improved considerably through advances in microbiological diagnostics, molecular detection techniques, infection prevention and control practices, and antimicrobial stewardship initiatives. However, biofilm-associated resistance and the continued emergence of antimicrobial resistance remain major therapeutic challenges [12-15].

## Review

Search strategy

**Figure 1:**
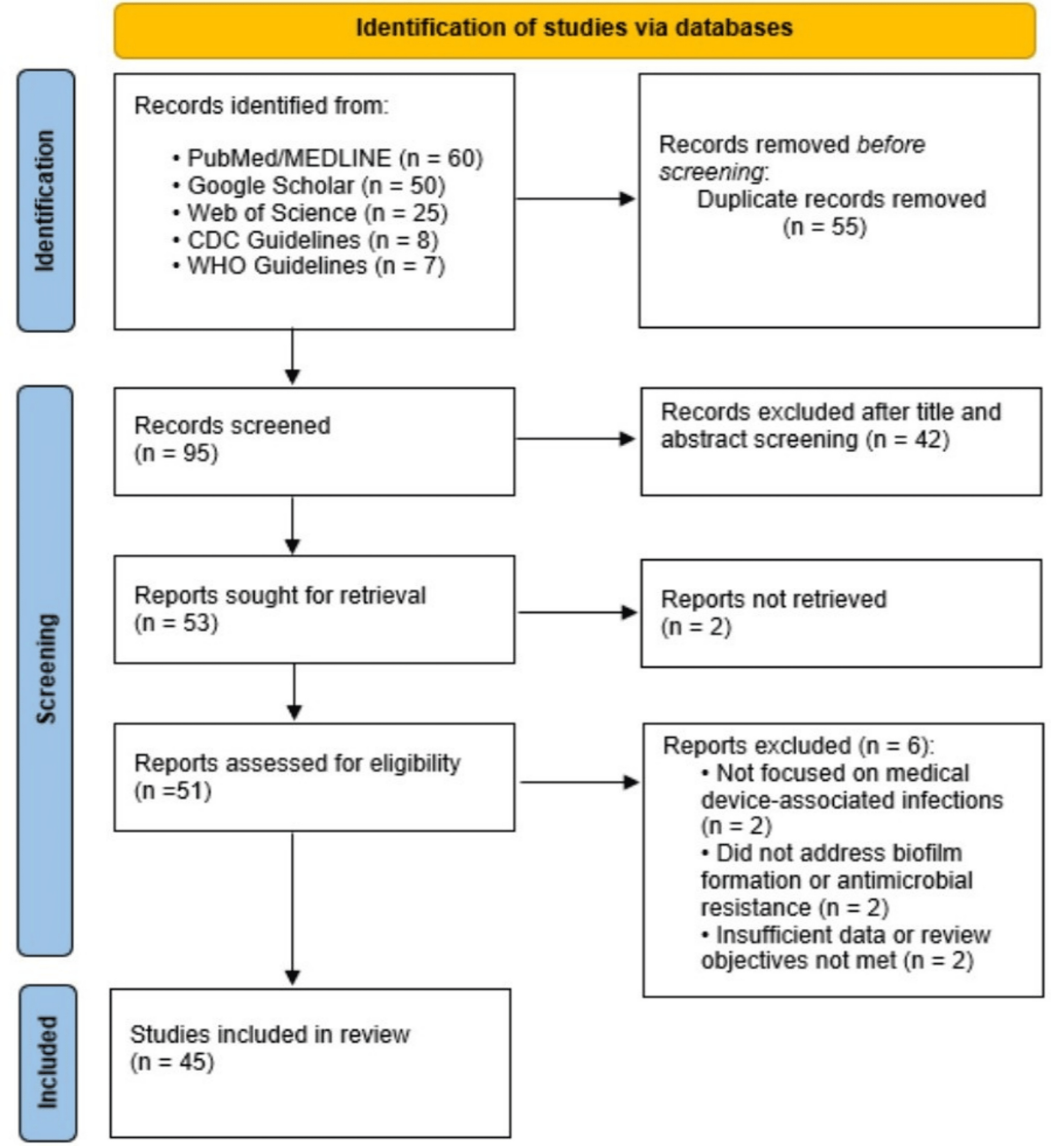
PRISMA flow diagram illustrating the study selection process for the review of medical device-associated infections, biofilm formation, and antimicrobial resistance. PRISMA: Preferred Reporting Items for Systematic Reviews and Meta-Analyses; CDC: Centers for Disease Control and Prevention; WHO: World Health Organization

The literature search was conducted using electronic databases, including Google Scholar, PubMed/MEDLINE, Scopus, and Web of Science, together with guideline documents from the Centers for Disease Control and Prevention (CDC) and the World Health Organization (WHO). Publications published between 1999 and 2024 were considered to capture both foundational studies and recent developments in the field. Medical Subject Headings (MeSH) terms and free-text keywords included “medical device-associated infections,” “healthcare-associated infections,” “catheter-associated urinary tract infections (CAUTI),” “central line-associated bloodstream infections (CLABSI),” “ventilator-associated pneumonia (VAP),” “biofilm,” “biofilm formation,” “biofilm resistance,” “multidrug resistance (MDR),” “extensively drug resistance (XDR),” “antimicrobial resistance,” “extended-spectrum β-lactamase (ESBL),” “carbapenem resistance,” and key pathogens such as *Staphylococcus aureus*, *Pseudomonas aeruginosa*, *Acinetobacter baumannii*, *Klebsiella pneumoniae*, and *Candida* species. These terms were combined using appropriate Boolean operators (AND/OR) to identify studies relevant to the objectives of the review. Studies addressing biofilm mechanisms, antimicrobial resistance patterns, device-associated infections, and the clinical or epidemiological characteristics of MDR/XDR pathogens were included. Clinical guidelines, surveillance reports, and international as well as regional studies, including those conducted in India, were also considered. Articles unrelated to medical DAIs, studies not focusing on biofilm formation or antimicrobial resistance, non-English-language publications, and articles without accessible full texts were excluded. A total of 150 records were identified through database and guideline searches, including PubMed/MEDLINE (n = 60), Google Scholar (n = 50), Web of Science (n = 25), CDC guidelines (n = 8), and WHO guidelines (n = 7). After the removal of 55 duplicate records, 95 studies remained for title and abstract screening. Following screening, 42 records were excluded, leaving 53 reports for full-text retrieval. Of these, two reports could not be retrieved, and 51 reports were assessed for eligibility. Subsequently, six reports were excluded because they were not focused on medical DAIs (n = 2), did not address biofilm formation or antimicrobial resistance (n = 2), or provided insufficient data to meet the review objectives (n = 2). Finally, 45 studies met the inclusion criteria and were included in the qualitative review. As this review was designed as a structured narrative review, no quantitative meta-analysis or statistical pooling of study outcomes was performed. The evidence was synthesized descriptively, and quantitative findings reported in the manuscript represent data extracted from the individual included studies rather than pooled statistical estimates. The study selection process is depicted in Figure 1.

The final analysis synthesized evidence from these sources to provide a comprehensive understanding of medical DAIs, with particular emphasis on biofilm formation and the growing challenge of antimicrobial resistance.

Brief scenario

A wide range of infections connected to invasive medical devices and procedures are referred to as HAIs. The most common kinds are catheter-associated urinary tract infections (CAUTIs), central line-associated bloodstream infections (CLABSIs), and ventilator-associated pneumonia (VAP). Among the most common hospital-acquired infections, CAUTIs raise medical expenses and increase patient morbidity [16,17]. Conversely, VAP and CLABSIs are linked to worse clinical outcomes and higher death rates [18,19]. In intensive care units, where patients frequently undergo prolonged catheterization and invasive procedures, DAIs are particularly prevalent [20,21]. Due in large part to insufficient infection control measures, the prevalence of these infections is even higher in developing nations [21-23].

Epidemiology and prevalence

Medical DAIs continue to be one of the most frequent problems experienced by hospitalized patients, especially in intensive care units, and constitute a significant fraction of HAIs globally. The majority of HAIs are associated with invasive medical devices, such as urinary catheters, central venous catheters, and mechanical ventilators, according to the CDC and the National Healthcare Safety Network (NHSN) [24]. About 30-40% of all hospital-acquired infections in hospitals are CAUTIs, which are the most common DAIs worldwide [25]. VAP and CLABSIs are linked to considerably higher mortality, longer intensive care unit stays, and higher medical costs [26]. The burden of HAIs differs across industrialized and developing nations, as large epidemiological surveys have shown. According to the WHO, overcrowding, insufficient surveillance systems, and a lack of infection control resources all contribute to the significantly greater occurrence of hospital-acquired infections in underdeveloped nations [27]. Comparing intensive care units from low- and middle-income countries to healthcare settings in wealthy countries, the International Nosocomial Infection Control Consortium (INICC) found significantly higher incidence of CAUTIs, CLABSIs, and VAP [28]. Numerous studies conducted in India have brought attention to the rising prevalence of DAIs in intensive care units and tertiary care institutions. Critically sick patients have a high rate of DAIs, especially in intensive care units where long-term catheterization and ventilator support are typical [29]. In a similar vein, surveillance studies conducted in India’s rural and urban healthcare facilities revealed that CAUTIs were the most prevalent medical DAI, followed by VAP and CLABSIs. The introduction of MDR and XDR infections further complicates the epidemiology of medical DAIs. Methicillin-resistant *Staphylococcus aureus* (MRSA), Enterobacteriaceae that produce ESBL, carbapenem-resistant *Acinetobacter baumannii*, and MDR *Pseudomonas aeruginosa* are among the organisms that are increasingly linked to device-related illnesses globally. In hospital settings, biofilm formation plays a major role in the persistence and spread of these resistant pathogens [30]. According to recent research, biofilm-producing bacteria are linked to poor treatment results, increased antibiotic resistance, recurring infections, and higher morbidity. Additionally, prolonged hospital stays, improper antibiotic use, and insufficient antimicrobial stewardship strategies have led to a sharp rise in the incidence of MDR/XDR bacteria in DAIs [31]. The usage of invasive medical devices, aging populations, and the growth of critical care services are all contributing factors to the ongoing global burden of medical DAIs. Therefore, to lower morbidity, mortality, and healthcare expenses related to these infections, ongoing surveillance, early diagnosis, and strict infection control measures are still crucial [32-36].

Major multidrug-resistant pathogens associated with medical device-related infections

Staphylococcus aureus

*Staphylococcus aureus* is one of the major pathogens responsible for bloodstream infections associated with catheter use and prosthetic medical devices. MRSA strains commonly exhibit resistance to β-lactam antibiotics, aminoglycosides, macrolides, and fluoroquinolones. In addition, biofilm-producing MRSA isolates demonstrate reduced susceptibility to vancomycin and linezolid. Methicillin resistance contributes to enhanced biofilm formation, increasing bacterial persistence on medical devices and complicating treatment outcomes [37].

Coagulase-Negative Staphylococci

Coagulase-negative staphylococci, particularly *Staphylococcus epidermidis*, are the most common cause of catheter-related infections. These organisms frequently exhibit resistance to methicillin, ciprofloxacin, gentamicin, and erythromycin. Their ability to form biofilms, largely mediated by slime production and the intercellular adhesion genes (*icaADBC*), plays a crucial role in antimicrobial resistance and prolonged colonization of indwelling medical devices [37].

Escherichia coli

*Escherichia coli* is one of the most frequently isolated pathogens in CAUTIs. ESBL-producing strains exhibit high levels of resistance to cefotaxime, ceftriaxone, ciprofloxacin, cotrimoxazole, and aminoglycosides. Furthermore, increasing carbapenem resistance has been reported due to the emergence of New Delhi metallo-β-lactamase (NDM)-mediated resistance mechanisms, posing significant therapeutic challenges [38].

Klebsiella pneumoniae

*Klebsiella pneumoniae* demonstrates extensive multidrug resistance through the production of ESBLs and carbapenemases. Biofilm-forming isolates show enhanced resistance to cephalosporins, carbapenems, aminoglycosides, and quinolones. Carbapenem-resistant *Klebsiella pneumoniae* has emerged as a major global public health concern due to its limited treatment options and high morbidity rates [39].

Pseudomonas aeruginosa

*Pseudomonas aeruginosa* is frequently associated with catheter-associated infections and VAP. Resistance to ceftazidime, ciprofloxacin, meropenem, and piperacillin-tazobactam is commonly observed. Its ability to form biofilms further enhances antimicrobial tolerance and facilitates long-term persistence in healthcare environments, making treatment particularly challenging [39].

Acinetobacter baumannii

*Acinetobacter baumannii* is considered one of the most problematic MDR pathogens associated with medical DAIs, especially in intensive care units. High rates of resistance to cephalosporins, aminoglycosides, fluoroquinolones, and carbapenems have been reported worldwide. The widespread occurrence of OXA-type carbapenemases and NDM enzymes contributes significantly to its extensive drug resistance, making infections caused by this organism difficult to treat and control [39].

Candida Species

In addition to bacterial pathogens, *Candida *species have emerged as important causes of medical DAIs, particularly in critically ill and immunocompromised patients. *Candida albicans* remains the most frequently isolated species; however, non-albicans *Candida species*, especially *Candida auris*, have gained increasing attention because of their ability to form biofilms on indwelling medical devices, persist in the hospital environment, and exhibit resistance to multiple antifungal agents. *Candida*-associated biofilms on central venous catheters, urinary catheters, and other implanted devices contribute to persistent bloodstream and urinary tract infections, often necessitating device removal in addition to antifungal therapy. Consequently, early diagnosis, appropriate antifungal treatment, and strict infection prevention and control measures are essential for reducing the morbidity and mortality associated with these infections.

Virulence and pathogenesis

The emergence of XDR and MDR pathogens, which worsen treatment outcomes, is a major challenge in managing DAIs. By restricting drug penetration, promoting horizontal gene transfer, and facilitating adaptive responses to stress, biofilms contribute to antimicrobial resistance. Common healthcare-associated pathogens such as *Staphylococcus aureus*, *Pseudomonas aeruginosa*, *Acinetobacter baumannii*, and* Klebsiella pneumoniae* frequently exhibit MDR and XDR phenotypes, making infections difficult to eradicate and often necessitating removal of the infected device [40]. The ability of microorganisms to adhere to medical device surfaces and form biofilms, organized microbial communities encased in a self-produced extracellular polymeric matrix, is the primary cause of the pathogenesis of DAIs. Because they act as barriers that prevent the penetration of antimicrobial agents and shield pathogens from the host immune system, these biofilms are essential for the persistence of infections [40]. This process is facilitated by several virulence factors, such as adhesins that allow attachment to both living and non-living surfaces, enzymes such as lipases and proteases that aid in tissue invasion, toxins that harm host tissues, and quorum-sensing mechanisms that regulate coordinated gene regulation and biofilm formation. Microorganisms exhibit altered metabolic activity and form specialized groups known as persister cells within biofilms. These cells contribute significantly to recurrent and persistent infections and are extremely resistant to antimicrobial therapy [40].

Clinical manifestations

The type of device, the pathogen, and the patient’s immune status all influence the clinical manifestations of medical DAIs. These infections typically present with localized signs such as redness, tenderness, warmth, swelling, and purulent discharge at the device insertion site, along with systemic manifestations including fever, chills, and leukocytosis [34]. Patients with CAUTIs may present with fever, dysuria, urinary urgency, and suprapubic discomfort; however, these symptoms may be subtle or absent, particularly in elderly or long-term catheterized patients. CLABSIs commonly manifest with fever, chills, and signs of sepsis and are frequently confirmed by positive blood cultures. VAP is characterized by fever, purulent tracheal secretions, worsening oxygenation, and new or progressive pulmonary infiltrates on chest imaging. Although this review primarily focuses on DAIs encountered in intensive care settings, prosthetic joint and other implant-associated infections also represent important medical DAIs and may present with chronic pain, inflammation, implant loosening, and, in some cases, sinus tract formation [35]. In addition to bacterial pathogens, *Candida* species, particularly in critically ill or immunocompromised patients, may cause DAIs involving central venous catheters and urinary catheters, often presenting with persistent bloodstream or urinary tract infections despite appropriate antimicrobial therapy. Biofilm formation on device surfaces promotes chronic and recurrent infections, which are frequently characterized by persistent low-grade inflammation and a poor response to antimicrobial therapy. Furthermore, infections caused by XDR and MDR organisms may present with more severe disease, prolonged hospitalization, and limited therapeutic options, thereby increasing morbidity, mortality, and healthcare costs [36]. Gram-positive cocci capable of biofilm formation and MDR Gram-negative bacilli remain the predominant bacterial causes of medical DAIs. Biofilm development further enhances antimicrobial resistance by limiting antibiotic penetration, facilitating horizontal gene transfer, and promoting the persistence of dormant bacterial cells within the biofilm matrix [35,36].

Antimicrobial resistance in medical device-associated infections

Biofilm formation and multidrug resistance are major contributors to the persistence and treatment failure of medical DAIs. Compared with planktonic organisms, biofilm-associated pathogens exhibit increased tolerance to antimicrobial agents and disinfectants, facilitating chronic and recurrent infections. Gram-negative pathogens commonly demonstrate high levels of resistance to quinolones, cephalosporins, aminoglycosides, and carbapenems. Several studies from India have reported resistance rates exceeding 90% for ciprofloxacin, more than 85% for cefepime, and over 80% for meropenem and piperacillin-tazobactam among device-associated isolates [39]. The emergence of MDR pathogens, particularly *Enterococcus faecium*, *Staphylococcus aureus*, *Acinetobacter baumannii*, *Klebsiella pneumoniae*, and *Pseudomonas aeruginosa*, has further complicated infection management in intensive care settings. The coexistence of biofilm formation and antimicrobial resistance significantly enhances pathogen survival, transmission, and persistence on medical devices, posing a substantial challenge to patient care and infection control programs. The major pathogens associated with medical DAIs, together with their common resistance patterns and important resistance mechanisms, are summarized in Table 1 [37-39].

**Table 1:** Common isolates and their resistance patterns and resistance mechanisms. MRSA: methicillin-resistant *Staphylococcus aureus*; CONS: coagulase-negative staphylococci; VRE: vancomycin-resistant *Enterococcus*; ESBL: extended-spectrum β-lactamase; KPC: *Klebsiella pneumoniae* carbapenemase; NDM: New Delhi metallo-β-lactamase; OXA: oxacillinase-type carbapenemase; XDR: extensively drug-resistant; *m*ecA: gene encoding penicillin-binding protein 2a (PBP2a); *ica*: biofilm-associated intercellular adhesion genes; *vanA/vanB*: genes associated with vancomycin resistance; PBP2a: altered penicillin-binding protein conferring methicillin resistance; *ERG11*: gene encoding lanosterol 14α-demethylase; MexB: efflux pump protein of *Pseudomonas aeruginosa*; OXA-23: carbapenemase enzyme of *Acinetobacter baumannii*

Organism	Common resistance pattern	Important resistance mechanisms
*Staphylococcus aureus* (MRSA)	Resistance to methicillin, penicillins, cephalosporins, fluoroquinolones	*mecA* gene, biofilm formation
CONS	Methicillin resistance, macrolide resistance	Slime production, *ica* genes
Enterococcus faecium	Vancomycin resistance (VRE), aminoglycoside resistance	*vanA/vanB* genes
Escherichia coli	Resistance to cephalosporins, quinolones, aminoglycosides	ESBL production
Klebsiella pneumoniae	Carbapenem resistance, ESBL production	KPC, NDM, OXA enzymes
Pseudomonas aeruginosa	Resistance to carbapenems, cephalosporins, quinolones	Efflux pumps, porin loss, biofilm
Acinetobacter baumannii	XDR, carbapenem resistance	OXA carbapenemases, biofilm
*Candida *spp.	Azole resistance	Biofilm-associated resistance

Detection of multidrug-resistant pathogens and biofilm formation in medical device-associated infections

Detection of MDR patterns in medical DAIs requires a comprehensive microbiological and epidemiological approach. Medical DAIs, including VAP, CLABSI, and CAUTIs, represent a significant proportion of HAIs and are associated with increased morbidity, mortality, prolonged hospital stays, and higher healthcare costs. Methods for the detection and surveillance of MDR pathogens in medical DAIs are summarized in Table 2 [1,27-30,36].

**Table 2:** Methods for detection and surveillance of MDR pathogens in medical device-associated infections. AST: antimicrobial susceptibility testing; CLSI: Clinical and Laboratory Standards Institute; MDR: multidrug-resistant; XDR: extensively drug-resistant; ESBL: extended-spectrum β-lactamase; DDST: double-disc synergy test; MDDST: modified double-disc synergy test; AmpC: AmpC β-lactamase; PCR: polymerase chain reaction; CFU/mL: colony-forming units per milliliter; CDC: Centers for Disease Control and Prevention; NHSN: National Healthcare Safety Network; CAUTI: catheter-associated urinary tract infection; CLABSI: central line-associated bloodstream infection; VAP: ventilator-associated pneumonia; BACTEC: automated blood culture detection system; VITEK-2: automated microbial identification and susceptibility testing system

Method	Technique/Tool used	Purpose	Key features
Sample collection and culture	Aseptic collection, culture on blood agar, MacConkey agar	Isolation of pathogens	Confirms infection using colony counts (e.g., ≥10⁵ CFU/mL)
AST	Kirby–Bauer disc diffusion (CLSI guidelines)	Determine resistance pattern	Classifies isolates as MDR/XDR based on resistance to multiple drug classes
Automated detection systems	BACTEC, VITEK-2	Rapid identification and susceptibility profiling	Reduces turnaround time, improves accuracy
ESBL detection	DDST, MDDST	Detect β-lactamase production	Shows synergy between cephalosporins and clavulanic acid; cefepime improves detection
AmpC detection	AmpC disc test	Detect AmpC β-lactamase enzyme	Identified by flattening/indentation of inhibition zone
Inhibitor-based detection	Clavulanic acid, sulbactam combination tests	Confirm ESBL production	≥5 mm increase in inhibition zone indicates ESBL positivity
Surveillance systems	CDC/NHSN guidelines, hospital surveillance programs	Monitor MDR/XDR trends	Tracks CAUTI, CLABSI, and VAP rates and resistance patterns
Biofilm detection/impact	Microtiter plate assay, slime detection methods	Identify biofilm-associated resistance	Biofilms increase antimicrobial resistance and persistence on devices
Molecular methods	PCR (limited use)	Detect resistance genes	Highly accurate but costly and less accessible
Advanced resistance detection	Carbapenemase detection	Identify XDR pathogens	Detects resistance in organisms like *Acinetobacter, Pseudomonas*

Biofilm detection is an important component of the microbiological evaluation of medical DAIs, as biofilm-forming organisms exhibit increased antimicrobial resistance, enhanced survival on device surfaces, and prolonged persistence within the host. Several phenotypic methods are routinely employed in clinical microbiology laboratories to identify and characterize biofilm production. The commonly used methods for biofilm detection and their key features are summarized in Table 3 [37].

**Table 3:** Methods for identification and characterization of biofilm formation. CRA: Congo red agar; TCP method: tissue culture plate method; OD: optical density; ELISA: enzyme-linked immunosorbent assay

Method	Principle	Procedure	Interpretation	Advantages	Limitations
Tube method	Detection of biofilm by staining adherent film on tube walls	Organism inoculated in broth → incubated → decanted → stained with crystal violet	Visible film lining wall and bottom of tube indicates biofilm production	Simple, low cost, easy to perform	Subjective interpretation, low reproducibility, qualitative only
CRA method	Detection based on colony morphology and color change on CRA	Organism cultured on CRA plates → incubated	Black, dry crystalline colonies = biofilm producer; red colonies = non-producer	Easy screening method, no special equipment	Low sensitivity and specificity, not quantitative
Microtiter plate assay (TCP method)	Quantitative measurement of adherent biofilm using optical density	Organism grown in microtiter plate → stained with crystal violet → OD measured using ELISA reader	Higher OD values indicate strong biofilm producers	Gold standard, quantitative, reproducible, sensitive	Requires spectrophotometer/ELISA reader, more time-consuming

Risk factors associated with medical device-associated infections

Numerous risk factors have an impact on the development of medical DAIs. Long-term use of invasive medical devices is a major contributing factor, as it provides a direct route for microorganisms to enter sterile body sites [4]. Longer device placement increases the risk of infection by encouraging microbial colonization and biofilm formation. Susceptibility to these infections is also increased by patient-related factors such as advanced age, immunocompromised conditions, and underlying comorbidities such as diabetes and chronic diseases [41]. The persistence of infections is largely dependent on microbial factors, particularly biofilm formation. Microorganisms are shielded from antimicrobial therapies and the host’s immune system by biofilms, which promote resistance and persistent infections [3,12,15]. Pathogens with strong antibiotic resistance, such as *Pseudomonas aeruginosa*,* Acinetobacter baumannii*, and* Staphylococcus aureus*, are commonly associated with biofilm-related infections [42]. Inadequate infection control procedures, cross-transmission of pathogens via healthcare personnel or contaminated environments, and incorrect aseptic techniques during device insertion and maintenance are all major contributing factors. Because of the frequent invasive procedures and increased exposure to resistant organisms, intensive care unit settings are especially high-risk. Furthermore, the emergence and spread of MDR/XDR pathogens are facilitated by the improper or overuse of broad-spectrum antibiotics [43].

Prevention and control of multidrug-resistant pathogens in medical device-associated infections

A comprehensive approach is needed to stop the spread of XDR and MDR pathogens, as well as medical DAIs. Strict adherence to infection control procedures, such as hand hygiene, aseptic device insertion methods, and proper device maintenance to lower infection risk, is an important precaution [35,34]. Infection rates are considerably reduced when devices are used for shorter periods of time and removed as soon as they are no longer required. By encouraging appropriate antibiotic use and reducing needless exposure to broad-spectrum medications, antimicrobial stewardship programs are crucial to preventing the development of resistance [44]. Healthcare facilities can identify high-risk areas and implement targeted interventions by routinely monitoring infection rates and resistance trends [20,30]. Additionally, reducing persistent infections requires preventing biofilm formation through antimicrobial-coated devices and efficient sterilization techniques [40-43]. Healthcare personnel receive ongoing education and training on infection prevention procedures, which enhances adherence and lowers the risk of transmission [44,45].

## Conclusions

Medical DAIs remain a significant challenge in healthcare due to the widespread use of invasive devices and the rise of MDR and XDR pathogens. These infections result from a complex interaction of host susceptibility, prolonged device use, microbial virulence, and lapses in infection control. A critical factor is biofilm formation, which enhances microbial survival and resistance, reducing the effectiveness of conventional antimicrobial treatments. Accurate and timely detection of MDR and XDR pathogens through culture, susceptibility testing, and identification of resistance mechanisms is crucial for guiding effective therapy. However, detection must be complemented by robust surveillance and continuous monitoring of resistance trends. The increasing antimicrobial resistance highlights the urgent need for prudent antibiotic use and strong antimicrobial stewardship programs. Prevention is key to controlling medical DAIs, requiring strict aseptic techniques, optimized device use, and evidence-based infection control measures. A multidisciplinary approach involving microbiological diagnostics, clinical management, and hospital infection control policies is essential for effective management and prevention.
